
JOURNAL INFORMATION
==============================
NLM Title Abbreviation: ScientificWorldJournal
Journal ID: ScientificWorldJournal
NLM Journal ID: 101131163
Journal ID: TSWJ

The Scientific World Journal

ISSN: 2356-6140
EISSN: 1537-744X
Publisher: Wiley

ARTICLE INFORMATION
==============================
PMCID: PMC6083843
PMID: 30147623
DOI: 10.1100/tsw.2001.243
Article ID: 172185
Article version: 1
Subjects: Short Report

Role of Transforming Growth Factor 1 in Lymphocyte Development and Death

Bommireddy Ramireddy *
Ormsby Ilona
Yin Moying
Doetschman Thomas C.
Department of Molecular Genetics, Biochemistry and Microbiology, University of Cincinnati College of Medicine, Cincinnati, OH 45267-0524, USA
*Ramireddy Bommireddy: bommirr@email.uc.edu
Publication date: 2001
Electronic publication date: 2001 Nov 9
Volume: 1
First page: 146
Last page: 146
Copyright: Copyright © 2001 Ramireddy Bommireddy et al.
Copyright year: 2001
License: This is an open access article distributed under the Creative Commons Attribution License, which permits unrestricted use, distribution, and reproduction in any medium, provided the original work is properly cited.
License URL: https://creativecommons.org/licenses/by/3.0/

Funding: National Institutes of Health HD26471 National Institutes of Health ES05652 National Institutes of Health CA84291 National Institutes of Health AR44059

==============================
Miami *Nature Biotechnology* Short Reports
*TheScientificWorld* (2001) 1 (S3), 146SR
ISSN 1532-2246; DOI 10.1100/TSW.2001.243

### ROLE OF TRANSFORMING GROWTH FACTOR β1 IN LYMPHOCYTE DEVELOPMENT AND DEATH

Ramireddy Bommireddy*, Ilona Ormsby, Moying Yin, and Thomas C. Doetschman

Department of Molecular Genetics, Biochemistry and Microbiology, University of Cincinnati College of Medicine, Cincinnati, OH 45267-0524, USA
* bommirr@email.uc.edu

**INTRODUCTION.** Transforming growth factor β1 (TGFβ1) is a polypeptide growth factor known to exert multiple functions during development and in the adult stage as well (1-2). TGFβ1 knockout mice are normal at the time of birth and do not exhibit any developmental defect. After one week of birth these mice start developing multifocal inflammatory lesions and eventually die around three weeks of age (3). Further studies revealed that T lymphocytes are the primary effectors in this phenotype (Doetschman, unpublished observation). During T cell development in the thymus, T cell progenitors undergo massive proliferation and around 95% of them undergo apoptosis. Positively selected $CD4^+CD8^+$ double positive T cells undergo thymic selection where cells that recognize self-antigens with high affinity are induced to undergo apoptosis (negative selection) (4-5). Any perturbations in the thymic education process might lead to export of self-reactive T cells to the periphery. In order to find the role of TGFβ1 in preventing the inflammation, we have studied the lymphocyte apoptosis and proliferation.

**METHODS.** Annexin-V apoptosis kit from BD-Pharmingen was used to detect apoptosis. Single cell preparations from thymus and spleen were prepared, RBCs were depleted using ammonium chloride lysis buffer, washed twice with chilled PBS and suspended in 1x binding buffer at $1x10^6$/ml. 5μl of PE labeled annexin-V (binds to phosphatidyl serine on apoptotic cells) and 5μl of 7-AAD (viability marker) added to 100μl of cell suspension, mixed gently and incubated at room temperature for 20 minutes in the dark. After the incubation 400μl of 1x binding buffer was added to the cell suspension and analyzed in a Beckman Elite flow cytometer.

Proliferation was measured using BrdU flow kit from BD-Pharmingen.

**RESULTS.** Analysis of apoptosis levels revealed no significant role for TGFβ1 in vivo (Fig. 1). Extent of apoptosis is varied from 10-20% in thymocytes and 20-30% in splenocytes irrespective of age, and TGFβ1 genotype. Analysis of proliferation of thymocytes revealed that T cells from $TGF\beta1^{-/-}$ mice are hyper responsive as assessed by tritiated thymidine and BrdU incorporation (results will be presented and discussed during poster presentation).

Apoptosis of Thymocytes and Splenocytes (Day 9)

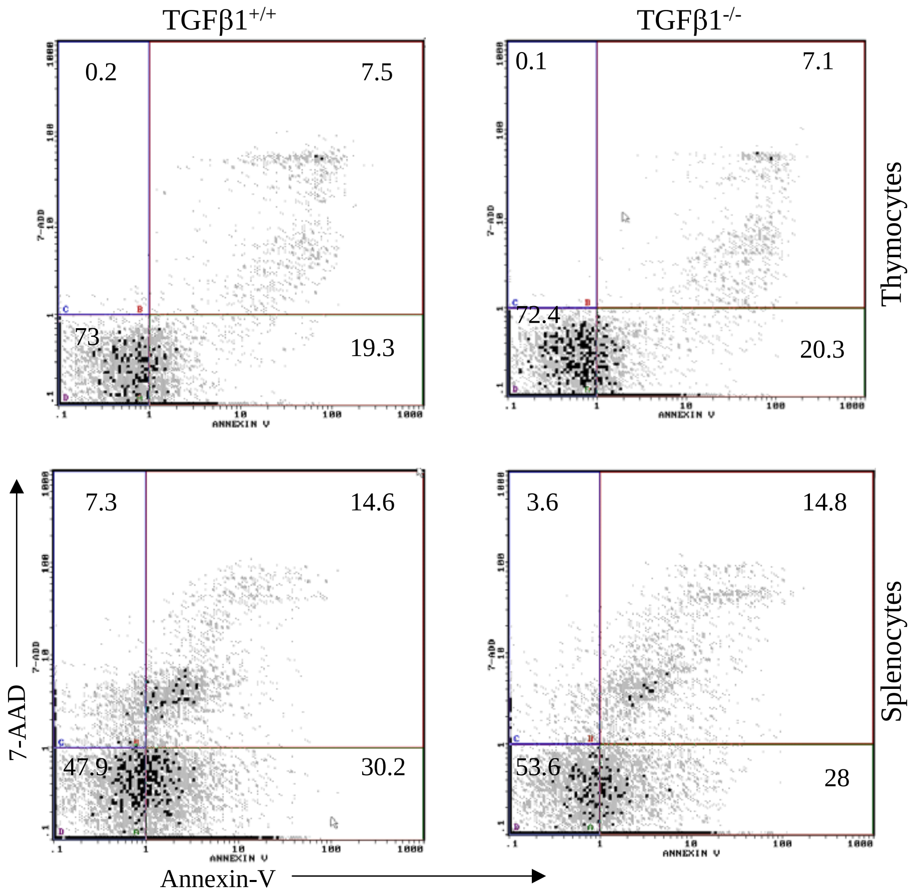

**Fig. 1**

**DISCUSSION.** Based on our observations, we propose that TGFβ1 doesn't prevent apoptosis. The minor differences observed between TGFβ1$^{+/+}$ and TGFβ1$^{-/-}$ lymphocytes are might be the result of hyperresponsiveness of TGFβ1$^{-/-}$ T cells. TGFβ1 negatively regulates lymphocyte proliferative responses. Altered activation signal threshold level of lymphocytes might lead to activation and accumulation of self-reactive T cells in the periphery.

**ACKNOWLEDGEMENT.** This work was supported by National Institutes of Health Grants HD26471, ES05652, CA84291, AR44059 (to T.C.D.)

**REFERENCES.**

1. Massague, J., Blain, S.W, and Lo, R.S. (2000) Cell 103, 295-309
2. Massague, J. (2000) Nat. Rev. 1, 169-178
3. Shull, M.M., Ormsby, I., Kier, A.B., Pawlowski, S., Diebold, R.J., Yin, M., Allen, R., Sidman, C., Proetzel, G., Calvin, D., et al. (1992) Nature 359, 693-699

4. Vasquez, N.J., Kaye, J., and Hedrick, S.M. (1992) J. Exp. Med. 175, 1307-1316
5. van Meerwijk, J.P., Marguerat, S., Lees, R.K., Germain, R.N., Fowlkes, B.J., and MacDonald, H.R. (1997) J. Exp. Med. 185, 377-383
6. Gong, Q., Cheng, A.M., Akk, A.M., Alberola-Ila, J., Gong, G., Pawson, T., and Chan, A.C. (2001) Nat. Immunol. 2, 29-36
